# Changes in Eicosanoid and Tumour Necrosis Factor-α Production by Rat Peritoneal Macrophages During Carrageenin-Induced Peritonitis

**DOI:** 10.1155/S0962935194000463

**Published:** 1994

**Authors:** W. M. Pruimboom, A. Verdoold, C. J. A. M. Tak, A. P. M. van Dijk, M. van Batenburg, J. H. P. Wilson, F. J. Zijlstra

**Affiliations:** 1 Dept of Pharmacology Erasmus University Rotterdam The Netherlands; 2 Dept of Internal Medicine II University Hospital Dijkzigt Rotterdam The Netherlands

## Abstract

Changes and correlations in cytokine and eicosanoid production by blood monocytes, non-purified and purified peritoneal cells during a carrageenin-induced peritonitis were investigated for a period of ten days. The cells were isolated and stimulated *in vitro*. Cytokine and eicosanoid production of the non-purified fraction increased steadily during peritonitis. During the whole episode of peritonitis the production capacity of granulocytes was very low and hardly any effect on the production capacity of macrophages (Mϕ) was observed. Cytokine and eicosanoid production of the non-purified fraction was mainly due to the presence of Mϕ. The production capacity of the peripheral blood monocytes was not similar to that of the peritoneal Mϕ.


Mediators of Inflammation 3, 335–340 (1994)

### Changes in eicosanoid and tumour necrosis factor-α production by rat peritoneal macrophages during carrageenin-induced peritonitis

**W. M. Pruimboom,[1,CA] A. Verdoold,[1] C. J. A. M. Tak,[1] A. P. M. van Dijk,[1] M. van Batenburg,[1] J. H. P. Wilson,[2] and F. J. Zijlstra[1]**

[1]Dept of Pharmacology, Erasmus University, Rotterdam and [2]Dept of Internal Medicine II, University Hospital Dijkzigt Rotterdam, The Netherlands

[CA] Corresponding Author

**CHANGES and correlations in cytokine and eicosanoid production by blood monocytes, non-purified and purified peritoneal cells during a carrageenin-induced peritonitis were investigated for a period of ten days. The cells were isolated and stimulated *in vitro*. Cytokine and eicosanoid production of the non-purified fraction increased steadily during peritonitis. During the whole episode of peritonitis the production capacity of granulocytes was very low and hardly any effect on the production capacity of macrophages (Mϕ) was observed. Cytokine and eicosanoid production of the non-purified fraction was mainly due to the presence of Mϕ. The production capacity of the peripheral blood monocytes was not similar to that of the peritoneal Mϕ.**

**Key words:** Eicosanoids, Peritoneal macrophages, Peritonitis, Tumour necrosis factor-α

### Introduction

Inflammation is the reaction to an injury such as an invasion by infectious agents. The blood supply to the inflamed area increases, venular permeability increases and leucocytes migrate out of venules into the surrounding tissue. At the site of an acute inflammation polymorphonuclear granulocytes (PMNs) produce large amounts of reactive oxygen intermediates, release a variety of hydrolytic enzymes and phagocytose pathogens.[1] By generating chemotactic factors, the migration of mononuclear cells (monocytes/Mϕ and lymphocytes) to the site of inflammation is stimulated.[2,3] During the chronic phase of the inflammatory response, Mϕ, mononuclear phagocytes derived from blood monocytes,[4] predominate over PMNs.[3] In addition to acting as a nonspecific defence, activated Mϕ[5,6] will initiate and control the specific defence by presenting processed antigens to lymphocytes[7] and by cytotoxity through direct cell–cell contact with tumour cells or infected cells,[8,9] during which they secrete cytokines and eicosanoids.[10,11] These inflammatory mediators also influence and regulate functions of other cells participating in the immune response.[12,13]

Cytokines are proteins with regulatory functions. They can induce other cytokines with overlapping effects and form interactive networks with hormones and eicosanoids. Mϕ are a potent source of a wide variety of cytokines,[14] whereas granulocytes are reported to produce only a limited spectrum of cytokines.[15–17]

Eicosanoids are bioactive lipids derived from arachidonic acid (AA) by cyclooxygenase and lipoxygenase pathways.[18,19] Eicosanoid production is species and tissue dependent, and often stimulus dependent.[20,21]

The aim of this work was to study cytokine (tumour necrosis factor-α, TNFα) and eicosanoid (leukotriene $B_4$, $LTB_4$; prostaglandin $E_2$, $PGE_2$; prostacyclin, detected as 6-keto-prostaglandin $F_{1\alpha}$, $6kPGF_{1\alpha}$; and thromboxane, $TXB_2$) production capacity of peritoneal PMNs, Mϕ and blood monocytes after the induction of peritonitis with a carrageenin solution, a sulfated polygalactan which stimulates cell-mediated immunity.[22] It was of interest to determine whether, during an episode of peritonitis, changes occurred in (a) the differentiation of influxed cells; (b) the cytokine and eicosanoid production capacity of blood monocytes and peritoneal cells; (c) interactions between different types of peritoneal cells concerning their production capacity; and (d) if there was a correlation between the production of eicosanoids and TNFα by blood monocytes and peritoneal Mϕ.

### Materials and Methods

*Animals and treatment*: Young male Wistar rats (13 weeks, approximately 200 g, six rats per group) were injected intraperitoneally with 2 ml of a carrageenin solution (Marine Colloids Inc., USA, 1 mg/ml) on day 0. Six rats received an injection of saline (control group).

© 1994 Rapid Communications of Oxford Ltd

*Cell isolation*: On day 1 (that is, 24 h after inducing the peritonitis with the carrageenin solution), three, seven and ten peritoneal cells and monocytes from the blood were isolated. With each group, cells were also isolated from a control animal, which was considered as day 0. Blood (± 7 ml) was collected in tubes with 1 ml EDTA (0.1 M) after decapitation. The blood was centrifuged (400 × ***g***, 4°C, 10 min) and from the leucocyte layer the monocytes were isolated into a 'monocyte' fraction by density gradient centrifugation using Percoll (d = 1.064 g/ml, Kabi–Pharmacia, Sweden; 400 × ***g***, 4°C, 25 min). Monocytes were washed three times with phosphate buffer solution (PBS, 400 × ***g***, 4°C, 10 min) and suspended in Dulbecco's modification of Eagle's medium (DMEM + HEPES (GIBCO, UK) + penicillin/streptomycin ($5 \times 10^4$ U/l per 50 mg/l, Flow Lab, UK) + foetal calf serum (10% FCS, GIBCO, UK) + L-glutamine (600 mg/l Flow Lab, UK), $1 \times 10^6$ cells/ml).

The peritoneal cavities were washed twice with 20 ml PBS (pH 7.4, 4°C, Oxoid, UK). The washings per rat were pooled, centrifuged (400 × ***g***, 4°C, 10 min) and suspended in 5 ml DMEM. $5 \times 10^6$ cells of this non-purified fraction were kept separate, the rest of this 'crude' fraction was separated on Percoll into a 'macrophage' and a 'granulocyte' fraction. These two purified fractions were washed three times with PBS (400 × ***g***, 4°C, 10 min), and suspended in DMEM ($1 \times 10^6$ cells/ml).

A small sample of cells from each fraction was stained by Hemacolor (Merck, Germany) and the different cell types were counted using a microscope (Zeiss, standard 25, Germany). The viability of the cells was determined by trypan blue exclusion.

*Cell incubation*: One million leucocytes per ml DMEM were plated on plastic culture dishes (Costar, UK). The cells were triggered for 15 min by calcium ionophore A23187 (Calbiochem, USA), 1 μM final concentration in dimethylsulfoxide (0.1% DMSO, Sigma, USA), 37°C, 7.5% $CO_2$). Controls were incubated with DMSO (0.1%).

The cells were also incubated for 24 h in the absence or presence of lipopolysaccharide (LPS, 10 μg/ml final concentration in PBS, LPS from *E. coli* 0111:B4 in PBS (Sigma, USA), 37°C, 7.5% $CO_2$). As a blank, PBS was added. At the end of the incubation the supernatant was centrifuged and kept at −80°C until required for analysis.

*Cytokine production*: TNFα production in the samples was determined directly in the supernatant by bioassay. For this bioassay the TNFα sensitive cell line WEHI-164 was used. The WEHI-164 cells were plated out in 96-well plates ($2 \times 10^4$ cells/50 μl/well, Costar, UK) and the samples (50 μl/well) or the human recombinant TNFα (hr-TNFα) standards (0.1–1000 u/ml hr-TNFα, 50 μl/well) were added. After 24 h incubation (37°C, 7.5% $CO_2$), MTT (tetrazolium salt, Sigma, USA) was added (0.125 mg/well) and after an incubation of 3 h the cells were lysed with buffer (20% sodium dodecyl sulfate (SDS) in 50% *N,N*-dimethylformamide (DMF), pH 4.7, 100 μl/well) for 18 h. The absorbance was measured at 595 nm with an ELISA reader (BIO-RAD, model 3550, UK). The TNFα production by the Mϕ was expressed as the cytotoxicity against WEHI-164 cells compared to the blank (DMEM complete).[23]

*Eicosanoid production*: Eicosanoid production ($LTB_4$, $PGE_2$, $TXB_2$ and $6kPGF_{1\alpha}$ from endogenous arachidonate of the samples was determined directly in the supernatant by radioimmuno assays (antibodies were obtained from Advanced Magnetics, USA; standards from Sigma, USA; and tritiated antigens from Amersham, UK). Cross-reactivities for individual antigens on antibodies were negligible.

*Statistical analysis*: Data are expressed as the mean ± standard error of the mean (S.E.M.). Data were analysed statistically with ANOVA followed by the Dunnett test or Student's *t*-test. The correlations were determined by the Pearson Correlation test. Data were considered significant when $p < 0.05$.

### Results

*Concentration and differentiation of cells obtained from the peritoneal cavity*: Induction of peritonitis in rats caused an increase of cells in the peritoneal cavity on day 1. The cell count dropped on the following days (Fig. 1A). A pronounced influx of granulocytes was seen on the first day which was followed by an influx of Mϕ, resulting in a Mϕ/granulocyte ratio of 4:1 from day 3 to 10 (Fig. 1B).

*Production capacity of cells to generate inflammatory mediators*: The inflammatory mediator production per million cells stimulated *in vitro* ($LTB_4$, 15 min A23187; and $PGE_2$, $TXB_2$, $6kPGF_{1\alpha}$, TNFα, 24 h LPS) by the peritoneal 'crude', 'macrophage' and 'granulocyte' fractions and the peripheral blood 'monocyte' fraction is shown in Fig. 2A, 2B, 2C and 2D.

Cytokine and eicosanoid production by the crude fraction increased steadily during peritonitis. TNFα and $TXB_2$ reached peak levels after 3 days, whereas $PGE_2$ and $6kPGF_{1\alpha}$ reached a plateau 7 days after induction of the peritonitis. In comparison with levels of other eicosanoids the $LTB_4$ production was low before and on the first day of the peritonitis and decreased significantly thereafter. By day 10 the production capacity returned to the initial level.

The production pattern of the cells from the macrophage fraction (Fig. 2B) was similar to that of cells from the crude fraction (Fig. 2A). Inflammatory mediators were also produced by the granulocyte fraction (Fig. 2C), although the production capacity

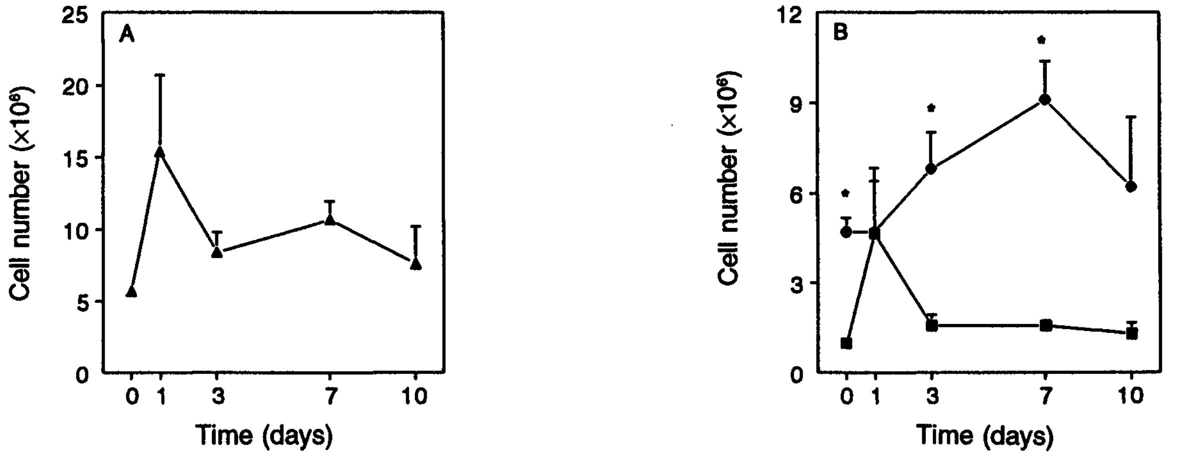

FIG. 1. Time course (0–10 days) of cell number (A) and differentiation of the cells (B) during a peritonitis in rats. Mϕ (●); granulocytes (■). Mean ± S.E.M.; $n = 6$ per group; $^{*}p < 0.05$ macrophages *vs.* granulocytes.

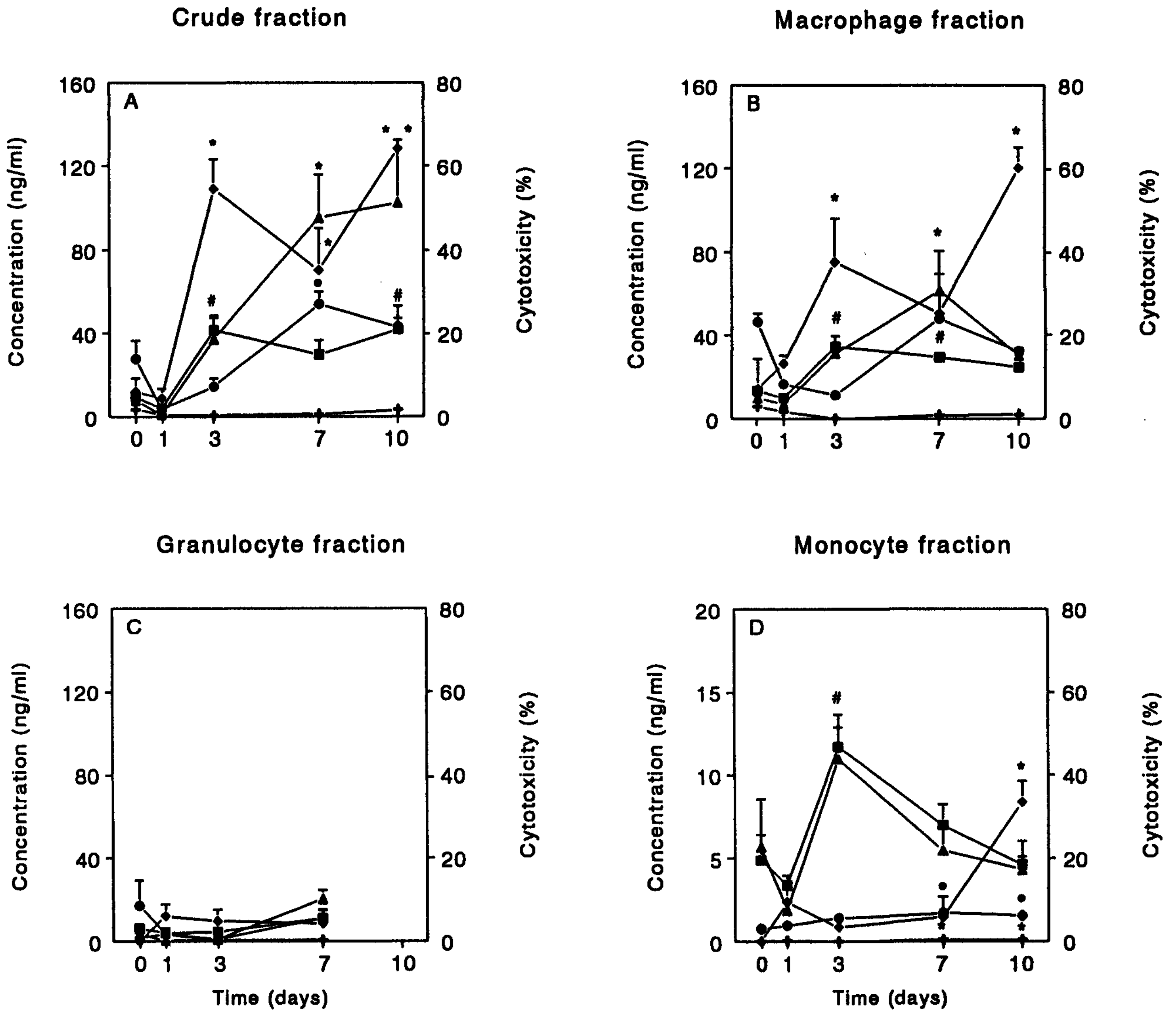

FIG. 2. Production capacity of stimulated 'crude' (A), 'macrophage' (B), 'granulocyte' (C) and 'monocyte' (D) fraction to produce the eicosanoids $LTB_4$ (+); $PGE_2$ (▲); $6kPGF_{1\alpha}$ (●); $TXB_2$ (■); and the cytokine TNFα (◆). $LTB_4$ production of $10^6$ cells/ml determined in supernatant after 15 min, 1 μM A23187 stimulation. $PGE_2$, $TXB_2$, $6kPGF_{1\alpha}$, TNFα production of $10^6$ cells/ml determined in supernatant after 24 h 10 μg/ml LPS stimulation; mean ± S.E.M.; $^{*,\#,\bullet}p < 0.05$ *vs.* day 0; $n = 4$–6.

of these cells was very low in comparison with cells from the crude and macrophage fraction. The pattern of inflammatory mediator production of the granulocyte fraction did not significantly change in time. On the tenth day after induction of the peritonitis, the concentration of the cells in the granulocyte fraction was too low to permit *in vitro* incubations.

The capacity of cells from the peripheral blood monocyte fraction to produce inflammatory mediators (Fig. 2D) was at least four times lower in comparison with both the crude and macrophage fractions. The production capacity of the cells from the monocyte fraction was about the same with or without stimulus. The patterns of the TNFα, $PGE_2$ and $6kPGF_{1\alpha}$ production of cells from the monocyte fraction were different from those of the macrophage and crude fraction. After induction of peritonitis the TNFα production of cells from the monocyte fraction increased significantly only on the tenth day, whereas $PGE_2$ and $TXB_2$ reached their highest level on the third day. The $6kPGF_{1\alpha}$ level did not change during peritonitis. The $LTB_4$ production was negligible.

*Correlations between inflammatory mediators produced in the same fraction*: In the crude fraction (Fig. 2A) there was a significant positive correlation between the production of $PGE_2$ and $6kPGF_{1\alpha}$ and $PGE_2$ and $TXB_2$ ($r = 0.6365$ and $r = 0.7145$). Similar significant correlations were found in the macrophage fraction (Fig. 2B, $r = 0.5580$ and $r = 0.6106$). In the monocyte fraction (Fig. 2D) the only significant positive correlation between the inflammatory mediators was between $PGE_2$ and $TXB_2$ ($r = 0.8881$).

*Correlation between inflammatory mediators in different fractions*: In Fig. 2A and 2B the patterns of inflammatory mediators produced by the stimulated crude and macrophage cell fractions are presented. There was always a significant positive correlation between these two fractions. The correlation varied from $r = 0.5671$ for $LTB_4$ to $r = 0.8586$ for TNFα. ($PGE_2$, $r = 0.6254$; $6kPGF_{1\alpha}$, $r = 0.7536$; and $TXB_2$, $r = 0.7502$). This correlation remained significant when we assumed that production of the cell inflammation mediators in the crude and macrophage fraction were only derived from the Mϕ (calculated to 100% macrophages, $LTB_4 = 0.4649$; $PGE_2 = 0.5855$; $6kPGF_{1\alpha} = 0.5506$; $TXB_2 = 0.7810$; and TNFα = 0.8674).

There were, however, some significant differences between the calculated (to 100% Mϕ) production levels of the crude and macrophage fractions. When separate days were considered the production levels of the calculated crude fraction in comparison with the calculated macrophage fraction was on day 1 lower for $PGE_2$ and $6kPGF_{1\alpha}$, and on day 3 higher for $LTB_4$, $TXB_2$ and TNFα (Fig. 3 A–E).

*Correlation coefficient between inflammatory mediators from monocyte and macrophage fraction*: Only the basal TNFα production of the cells from the monocyte and macrophage fraction were clearly correlated ($r = 0.5421$).

### Discussion

Characterization of inflammatory mediators produced by granulocytes and monocytes/macrophages during induced inflammation in animal models, may be helpful to the understanding of inflammatory diseases and their treatment. The present study investigated the changes and correlations in cytokine and eicosanoid production capacity of peripheral blood monocytes, non-purified ('crude' fraction) and purified ('macrophage' and 'granulocyte' fraction) peritoneal cells during a carrageenin-induced peritonitis in rats.

In the present study, where an influx of leucocytes into the peritoneum was achieved after carrageenin injection, the number of PMNs increased in the acute phase (day 1) and thereafter decreased quickly. On day 0, before inducing the peritonitis, there was a reasonable number of Mϕ present in the peritoneal cavity. The number of Mϕ increased from day 1 until 7 days after induction of the peritonitis, resulting in a mainly Mϕ cell population. Previous histological studies had similar results,[24,25] although a quantitative and kinetic method showed that both monocytes and PMNs from rat migrate rapidly to the inflamed site, the migration of PMNs, however, also declined long before the migration of monocytes started to decrease.[3] The pattern of this migration can be explained with the function of the leucocytes in inflammation. In the initial phase both PMNs and Mϕ are involved in nonspecific defence mechanisms.[1,2] In addition, Mϕ will also initiate and control the specific defence.[7–13] The mechanism of this selective migration is not clear. Leucocyte adhesion to the blood vessel endothelium, which is followed by transendothelial migration, is a multistep process in which several adhesion molecules are involved.[26,27]

Our experiment showed that the inflammatory mediator production capacity of the non-purified fraction, which was harvested from the inflamed peritoneal cavity, changed with time. The production of inflammatory mediators in the non-purified fraction was mainly caused by the presence of Mϕ during carrageenin-induced peritonitis. This is, first of all, based on the significant correlation between the eicosanoid and TNFα production capacity of the macrophage fraction and the crude fraction, these correlations hardly changed when it was assumed that these fractions only contained Mϕ. Secondly, the production capacity of the granulocytes appeared to

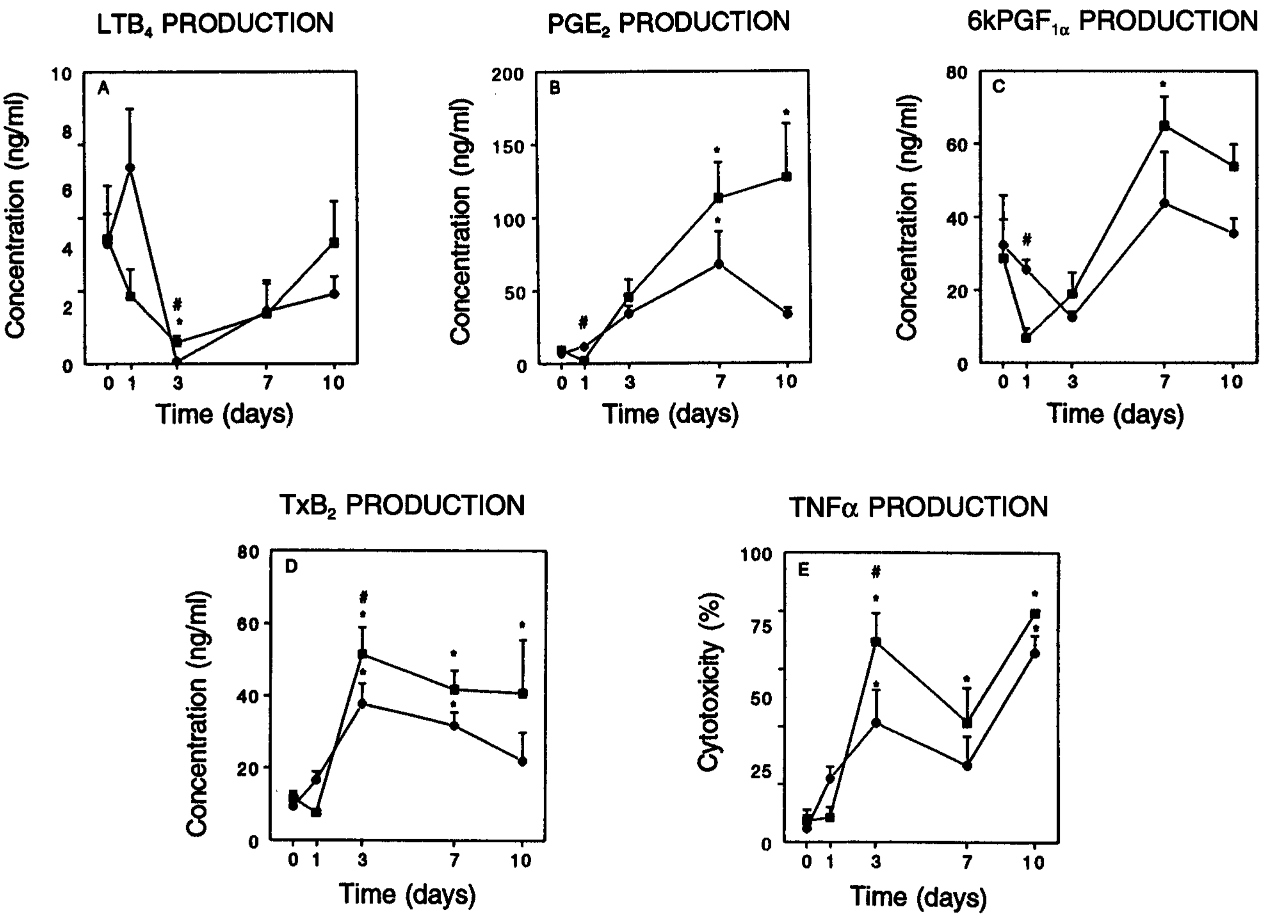

FIG. 3. Production capacity of the stimulated 'crude' (■) and 'macrophage' fraction (●) (calculated to 100% Mϕ) to produce the eicosanoids $LTB_4$ (A), $PGE_2$ (B), $6kPGF_{1\alpha}$ (C), $TXB_2$ (D) and the cytokine TNFα (E). $LTB_4$ production of $10^6$ cells/ml determined in supernatant after 15 min, 1 μM A23187 stimulation. $PGE_2$, $TXB_2$, $6kPGF_{1\alpha}$, TNFα production of $10^6$ cells/ml determined in supernatant after 24 h 10 μg/ml LPS stimulation; mean ± S.E.M.; $^*p < 0.05$ *vs.* day 0; $^\# p < 0.05$ granulocytes *vs.* macrophages; $n = 4–6$.

be very low. Moreover the production capacity of the granulocyte fraction did not correlate with the crude fraction.

When the production levels of the non-purified fraction was compared with the macrophage fraction, assuming that the inflammatory mediators were only produced by Mϕ, it was observed that the production of $PGE_2$ and $6kPGF_{1\alpha}$ had decreased on day 1 and the production of $LTB_4$, $TXB_2$ and TNFα had increased on day 3. The results of these effects could be an amplification of the inflammation, due to the decrease of anti-inflammatory substances and the subsequent increase of pro-inflammatory substances.

The low production capacity of the crude fraction in the acute phase followed by an increasing production capacity in the chronic phase could also be explained by presence of the Mϕ. Blood monocytes and resident Mϕ transform from primed into active Mϕ at the side of the inflammation, after which profound changes occur in morphology and function of these cells.[4–6] The Mϕ present at day 1 in the peritoneal cavity, probably were not primed *in vivo* at this time, which could be the reason that the eicosanoid and cytokine production capacity of these Mϕ were so low after stimulation *in vitro* with LPS. Once these Mϕ were primed *in vivo*, also the new monocytes that migrate into the peritoneal cavity, the production capacity of these monocytes/Mϕ steadily increased, showing *in vitro* a differentiated pattern for the eicosanoids and TNFα. This pattern probably depends on the function of the metabolite.

As peripheral blood monocytes transform into peritoneal Mϕ,[4] the correlation between these cells was investigated. The eicosanoid and TNFα production capacity of blood monocytes was not similar to that of Mϕ during peritonitis. It was clearly shown that through influx and transformation of monocytes into Mϕ the synthesis of mediators completely changed.

In conclusion, Mϕ are the main source of the inflammatory mediators whose production is dependent on the episode of the carrageenin-induced peritonitis.Characterization of purified Mϕ *in vitro* in animal and human models will be helpful to the understanding of inflammatory diseases and their treatment. Peripheral blood monocytes do not reflect mediator production of Mϕ present at the inflammatory site.

### References

1. Malech HL, Gallin JI. Neutrophils in human diseases. *New Engl J Med* 1987; **317:** 687–694.
2. Ternowitz T. Monocyte and neutrophil chemotaxis in psoriasis. *Dan Med Bull* 1989; **36:** 1–14.
3. Issekutz AC, Issekutz TB. Quantitation and kinetics of blood monocyte migration to acute inflammatory reactions, and IL-1α, tumor necrosis factor-α and IFN-gamma. *J Immunol* 1993; **151:** 2105–2115.
4. Van Furth R. The origin and kinetics of mononuclear phagocytes. *J Exp Med* 1968; **128:** 415–433.
5. Dougherty GJ, McBride WH. Macrophage heterogeneity, review. *J Clin Lab Immunol* 1984; **14:** 1–11.
6. Hamilton TA, Adams DO. Molecular mechanisms of signal transduction in macrophages. *Immunol Today* 1987; **8:** 151–158.
7. Unanue ER, Allen PM. The basis for the immunoregulatory role of macrophages and other accessory cells. *Science* 1987; **236:** 551–557.
8. Ben-Efraim S, Tak CJAM, Fieren MJWA, Romijn JC, Beckmann I, Bonta IL. Activity of human peritoneal macrophages against a human tumor: role of tumor necrosis factor-α, $PGE_2$ and nitrite, *in vitro* studies. *Immunol Lett* 1993; **37:** 27–33.
9. Groscurth P. Cytotoxic effector cells of the immune system (review). *Anat Embryol* 1989; **180:** 109–119.
10. Stein M, Keshav S. The versatility of macrophages. *Clin Exp Aller* 1992; **22:** 19–27.
11. Bonta IL, Ben-Efraim S. Interactions between inflammatory mediators in expression of antitumor cytostatic activity of macrophages. *Immunol Lett* 1990; **25:** 295–302.
12. Nathan CF. Secretory products of macrophages. *J Clin Invest* 1987; **79:** 319–326.
13. Cerami A. Inflammatory cytokines. *Clin Immunol Immunopath* 1992; **62:** S3–S10.
14. Balkwill FR, Burke F. The cytokine network. *Immunol Today* 1989; **10:** 299–303.
15. Lindemann A, Riedel D, Oster W *et al.* Granulocyte/macrophage colony-stimulating factor induces interleukin-1 production by human polymorphonuclear neutrophils. *J Immunol* 1988; **140** 837–839.
16. Cicco NA, Lindemann A, Content J, *et al.* Inducible production of interleukin-6 by human polymorphonuclear neutrophils: role of granulocyte–macrophage colony-stimulating factor and tumor necrosis factor-alpha. *Blood* 1990; **75:** 2049–2052.
17. Mandi Y, Endrész V, Krenács L, Régely K, Degré M, Béládi I. Tumor necrosis factor production by human granulocytes. *Int Allergy Appl Immunol* 1991; **96:** 102–106.
18. Ford-Hutchonson AW. Leukotrienes: their formation and role as inflammatory mediators. *Fed Proc* 1985; **44:** 25–29.
19. Samuelsson B, Goldyne M, Granström E, Hamberg M, Hammarström S, Malmsten C. Prostaglandins and thromboxanes. In: Snell EE, Boyer PD, Meister A, Richardson CC, eds. *Annual review of biochemistry.* California: Annual Previews Inc, 1978; 995–1029.
20. Ouwendijk RJTh, Zijlstra FJ, Broek van den AMWC, Brouwer A, Wilson JHP, Vincent JE. Comparison of the production of eicosanoids by human and rat peritoneal macrophages and Kupffer cells. *Prostaglandins* 188; **35:** 437–446.
21. Yoss EB, Spannhake EWm, Flynn JT, Fish JE, Peters SP. Arachidonic acid metabolism in normal human alveolar macrophages: stimulus specificity for mediator release and phospholipid metabolism, and pharmacologic modulation *in vitro* and *in vivo*. *Am J Respir Cell Mol Biol* 1990; **2:** 69–80.
22. Cochran FR, Baxter CS. Carrageenin-induced suppression of T-lymphocyte proliferation in the rat: abrogation of suppressor factor production by the prostaglandin synthesis inhibitors, indomethacin and ETYA. *Immunobiol* 1984; **166:** 275–285.
23. Garrelds I, Zijlstra FJ, Tak CJAM, Bonta IL, Beckmann I, Ben-efraim S. A comparison between two methods for measuring tumor necrosis factor in biological fluids. *Agents and Actions* 1993; Special conference issue: C89–C91.
24. Hurley JV, Ryan GB, Friedman A. The mononuclear response to inrapleural injections in the rat. *J Path Bact* 1966; **91:** 575–587.
25. Migliorisi G, Folkes E, Pawloski N, Cramer EB. *In vitro* studies of human monocyte migration across endothelium in response to leukotriene $B_4$ and f-Met–Leu–Phe. *Am J Pathol* 1987; **127:** 157–167.
26. Mackay CR, Imhof BA. Cell adhesion in the immune system. *Immunol Today* 1993; **14:** 99–102.
27. Springer TA. Adhesion receptors of the immune system. *Nature* 1990; **346:** 425–434.

**Received 28 March 1994; accepted in revised form 2 May 1994**
